# Illusory body ownership of an invisible body interpolated between virtual hands and feet via visual-motor synchronicity

**DOI:** 10.1038/s41598-018-25951-2

**Published:** 2018-05-15

**Authors:** Ryota Kondo, Maki Sugimoto, Kouta Minamizawa, Takayuki Hoshi, Masahiko Inami, Michiteru Kitazaki

**Affiliations:** 1 Department of Computer Science and Engineering, Toyohashi University of Technology, 1-1 Hibarigaoka, Tempaku, Toyohashi, Aichi 441-8580 Japan; 2 Faculty of Science and Technology, Keio University, 3-14-1 Hiyoshi, Kohoku-ku, Yokohama, Kanagawa 223-8522 Japan; 3 Graduate School of Media Design, Keio University, 4-1-1 Hiyoshi, Kohoku-ku, Yokohama, Kanagawa 223-8526 Japan; 4 Research Center for Advanced Science and Technology, The University of Tokyo, 4-6-1 Komaba, Meguro-ku, Tokyo, 153-8904 Japan

## Abstract

Body ownership can be modulated through illusory visual-tactile integration or visual-motor synchronicity/contingency. Recently, it has been reported that illusory ownership of an invisible body can be induced by illusory visual-tactile integration from a first-person view. We aimed to test whether a similar illusory ownership of the invisible body could be induced by the active method of visual-motor synchronicity and if the illusory invisible body could be experienced in front of and facing away from the observer. Participants observed left and right white gloves and socks in front of them, at a distance of 2 m, in a virtual room through a head-mounted display. The white gloves and socks were synchronized with the observers’ actions. In the experiments, we tested the effect of synchronization, and compared this to a whole-body avatar, measuring self-localization drift. We observed that visual hands and feet were sufficient to induce illusory body ownership, and this effect was as strong as using a whole-body avatar.

## Introduction

Illusory body ownership^1–3^ can be induced to a virtual body by visual-tactile contingent stimulation^4–22^ or visual-motor congruent actions^23–26^. The Rubber Hand Illusion is representative of visual-tactile-stimulation induced illusory body ownership. Stroking a participant’s hand and a rubber hand with paintbrushes at the same time causes illusory body ownership of the rubber hand if the participant sees only the rubber hand and paintbrush^4,6^. Virtual Reality systems have often been used for induction of visual-motor-contingent body ownership. When visual body movements are presented using a head-mounted display (HMD) and are synchronized with a participant’s actual body movements, he/she feels the virtual body as his/her own body^23,24^. The methods to cause illusory body ownership can be categorized into *passive* contingent visual-tactile stimulation and *active* synchronicity of visual body stimuli and motor actions. The active method induces a sense of agency in addition to body ownership and generally induces stronger body ownership than the passive method^27^.

The conscious experience of ownership of body parts such as the Rubber Hand Illusion^4–6,12,13,15–19,22,24,28–31^ and the experience of global ownership such as Full-Body Ownership^7,10,14,20,21,23,25–27,32–35^ should be considered separately to understand self-consciousness^1^. The studies of Full-Body Ownership contribute to investigate the idea of “minimal phenomenal selfhood”, that is, the conscious experience of being a self, and relate to the embodiment and the simplest form of self-consciousness^1^. The out-of-body experience has been investigated in neurological and clinical studies^36–38^. During an out-of-body experience, a person has the feeling of seeing their own body and the environment from a viewpoint that is distant from the physical body. It has been observed that brain damage and stimulus to the temporo-parietal junction can induce the out-of-body experience. Thus, the temporo-parietal junction is a critical region for the conscious experience of the normal self and its embodiment^39^.

Out-of-body experiences can be linked to illusory body ownership using passive visual-tactile stimulation^7–10^. Lenggenhager *et al*.^7^ presented a virtual body in front of the participant, and visually synchronized tactile sensation to his/her back to induce the full-body ownership illusion. The illusory body ownership of the virtual body caused the participant’s proprioceptive self-localization to drift toward the virtual body^7,11^. Pomés and Slater^10^ replicated the study of Lenggenhager *et al*.^7^ by measuring behavioral responses to a threat to a virtual body and included a questionnaire on the proprioceptive drift. They found significant perceptions of both a participant’s own body drifting toward the virtual body placed in front, and the virtual body moving backward in the synchronous condition. A significant positive correlation was observed between the feeling of illusory-body drift forward and responses to the threat, although the feeling of illusory body ownership and response to the threat were not significantly different between the synchronous and asynchronous conditions. Thus, the proprioceptive drift forward is associated with a greater response to the threat, while the feeling that the virtual body is moving backward decreases the response to the threat.

The proprioceptive drift of own body-part location was originally reported in the Rubber-Hand-Illusion studies^4,6^. Thus, the drift of proprioceptive self-body or body-part location has been considered as one of the behavioral measurements of illusory body ownership. However, it is reported that proprioceptive drift depends on the duration of visual-tactile sensations; the drift occurs with synchronous, asynchronous, or no tactile stimulation using short and frequent stimulations, and is prevented only by continuous exposure to asynchronous stimulation^12^. Thus, the feeling of ownership cannot be measured by the proprioceptive drift alone.

Body ownership can be induced in a wide variety of bodies^16,28,32–34^ or still objects^5^. Various studies have investigated how the experience of body ownership to different bodies changes human behavior and implicit social attitudes^18,25,26,35,40^. Illusory body ownership in different skin colors decreases implicit racial bias^18,25^. Adults’ illusory body ownership to a child body avatar modulates child-like implicit attitudes as well as object-size perception^26^. Thus, illusory body ownership can be induced to various bodies in different shapes, colors, and ages. In passive visual-tactile contingent stimulations, the synchronicity of visual and tactile stimuli is critical to induce such illusions, while in active visual-motor stimulations, the synchronicity of visual stimuli and motor action is critical.

Recently, it has been reported that body ownership can be induced to an empty space by presenting visual-tactile stimuli^17,20,21^. An entire invisible body ownership is induced when participants observe a paintbrush moving in an empty space and by defining the contours of an invisible body through an HMD from a first-person perspective while receiving simultaneous touches on the corresponding parts of their real body. The illusory ownership of an entire invisible body reduces autonomic and subjective social anxiety responses caused by standing in front of an audience^20^. In contrast, an illusion of missing body parts through illusory ownership of an amputated virtual body can be induced by eliminating a virtual (visual) body part and not applying physical touches to the body part corresponding to the missing part^22^. This illusory experience of amputation decreases corticospinal excitability of the illusory amputated body part.

The purpose of our study was to test whether the illusory ownership of an invisible body could be induced by the active method of visual-motor synchronicity, and if the illusory invisible body could be experienced in front of the observer similar to the full-body ownership illusion.

**Figure 1:**
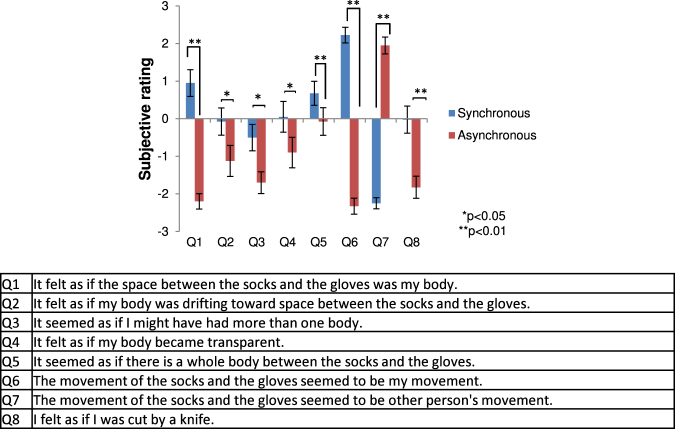
Results of Experiment 1. Subjective ratings of questionnaires. The error bars indicate SE.

**Figure 2:**
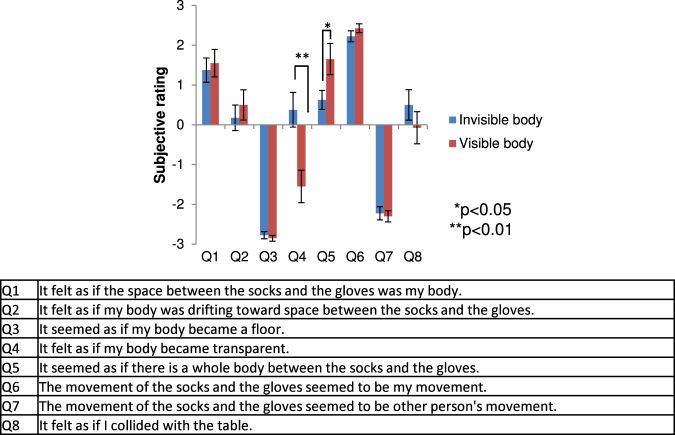
Results of Experiment 2. Subjective ratings of questionnaires. The error bars indicate SE.

In Experiment 1, we tested whether illusory body ownership can be induced by presenting only visual gloves and socks in synchrony and consistent with the observer’s own movements. The gloves and socks were presented in front of and facing away from the observers, in third-person perspective. We compared the synchronous condition, i.e. the virtual gloves and socks moved synchronously with the observer’s action, with the asynchronous condition, i.e. the gloves and socks moved independently of the observer’s action. In Experiment 2, we compared the invisible condition, i.e. where only gloves and socks were presented, with the visible body condition so that a whole-body avatar was presented. The whole-body avatar was also presented in front of and facing away from the observers. In these experiments, after participants moved their own body by observing the avatar stimuli for 5 min, a threat stimulus appeared suddenly (see the Methods section for details). Then, participants answered a questionnaire (see Figs 1 and 2). Finally, in Experiment 3, we tested whether self-localization drift could occur with illusory body ownership induced by only visual gloves and socks. When illusory body ownership occurs with the virtual invisible body in front of the participant, self-location will drift toward the virtual invisible body similar to the full-body ownership illusion^7^. All experiments were conducted in within-group designs where all subjects (20, 20, and 10 naïve participants for Experiment 1, 2, and 3, respectively) performed all conditions (synchronous vs asynchronous conditions in Experiment 1 and 3, and visible and invisible bodies in Experiment 2).

## Results

### Experiment 1

Participants (n = 20) rated the illusory body ownership higher when the virtual gloves and socks moved synchronously with their own movements than the asynchronous condition (Q1 in Fig. 1). The feeling of proprioceptive drift toward the invisible body was higher in the synchronous than the asynchronous condition (Q2). They felt as if their own body became transparent (Q4) more in the synchronous than the asynchronous condition. However, the result of Q4 (transparency) in the synchronous condition was approximately 0 (neutral) so the feeling of a transparent body was not obviously stronger relative to the asynchronous condition, although participants did perceive the illusory (invisible) body between the gloves and the socks (Q5) more strongly in the synchronous than the asynchronous condition. Overall, participants did not feel as if they were cut by the knife that suddenly appeared in the asynchronous condition (Q8); although the response in the synchronous condition was higher than the asynchronous condition, its score was approximately 0 (neutral).

These findings were supported by statistical tests, where the Wilcoxon signed-rank test indicated that the ratings of seven questions were significantly higher in the synchronous condition than the asynchronous condition. The probability of superiority of dependent measures (PS_dep_) indicated the effect size. The findings were; (Q1 [body ownership]: z = 3.84, p < 0.0001, PS_dep_ = 0.95; Q2 [proprioceptive drift]: z = 1.96, p = 0.050, PS_dep_ = 0.6; Q3 [multiple bodies]: z = 2.41, p = 0.015, PS_dep_ = 0.65; Q4 [transparent body]: z = 2.23, p = 0.024, PS_dep_ = 0.6; Q5 [illusory body perception]: z = 3.12, p = 0.001, PS_dep_ = 0.75; Q6 [synchronous movement]: z = 3.94, p < 0.0001, PS_dep_ = 1; Q8 [body cut]: z = 3.45, p < 0.0001, PS_dep_ = 0.85). The rating of Q7 [asynchronous movement] was significantly higher in the asynchronous condition than the synchronous condition (Q7: z = −3.93, p < 0.0001, PS_dep_ = 1). The participants answered that the gloves and socks moved synchronously with their actions in the synchronous condition (Q6) and moved asynchronously in the asynchronous condition (Q7).

### Experiment 2

The results of the experiments (n = 20) did not indicate statistical differences between the invisible body condition, i.e. only the gloves and socks were presented, and the visible body condition, i.e. a whole-body avatar was presented, in any questions except for Q4 [transparent body] (z = 3.42, p < 0.001, PS_dep_ = 0.75) and Q5 [illusory body perception] (z = −1.98, p = 0.048, PS_dep_ = 0.75; Fig. 2 left). As there were no differences in the feelings of body ownership (Q1: z = −0.77, p = 0.459, PS_dep_ = 0.6) and proprioceptive drift (Q2: z = −1.13, p = 0.283, PS_dep_ = 0.55), the illusory body ownership of the invisible body seems equivalent to the visible body. However, the visible avatar was not perceived as transparent, and was more clearly perceived as a whole body rather than an invisible body.

Q3 [Body is floor] was a control question to check random responses, and overall scores were close to the minimum value of −3, irrespective of the visibility condition. The participants answered that the virtual stimuli moved synchronously with their actions irrespective of the visibility condition because the virtual stimuli were synchronized with the participants’ actions in all trials of Experiment 2 (Q6 [synchronous movement]: z = −1.3696, p = 0.212, PS_dep_ = 0.5; Q7 [asynchronous movement]: z = 0.26, p = 0.826, PS_dep_ = 0.3).

The scores of Q8 [collision with a table] were generally 0, irrespective of the visibility condition (z = 1.49, p = 0.151, PS_dep_ = 0.5). Thus, the feeling of threat was not different between the visible and invisible conditions, and feelings were not strong or clear.

### Experiment 3

**Figure 3:**
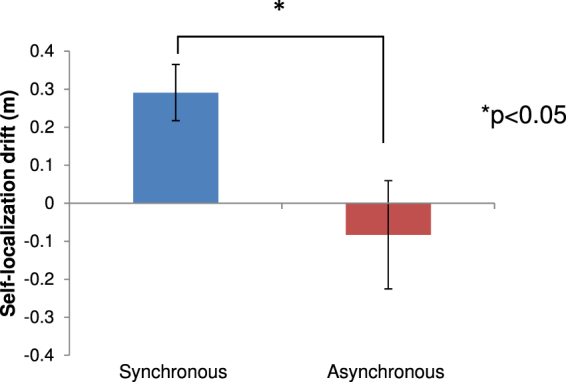
Results of Experiment 3. Drifts in the self-localization task.

We found that the proprioceptive self-location drifted forward more clearly in the synchronous condition than the asynchronous condition (n = 10, t(9) = 3.101, p = 0.013, d = 0.98; Fig. 3). Thus, proprioceptive self-location drift to the invisible body was perceived only for the gloves and socks.

## Discussion

We tested whether body ownership could be induced to an invisible body using virtual socks and gloves synchronized with a participant’s movement. We evaluated body ownership by subjective ratings and the self-localization task. We found that in the body ownership induced by only socks and gloves, observers perceived a complete body between socks and gloves, and the proprioceptive self-localization drift toward the invisible body was similar to the one observed in the full-body ownership illusion^7^.

In both Experiments 1 and 2, the feelings of threat to the invisible body and the visible body were not clear, even in the synchronous condition. This may have been caused by the weak illusory body ownership; the score of body ownership was 0.95 (of 3.0 maximum) for the invisible body in Experiment 1, 1.38 for the invisible body and 1.55 for the visible body in Experiment 2. In the experiments, the exposure time for the visual-motor synchronicity was 5 min. Prolonged exposure may enhance illusory body ownership and feelings toward the threat. Furthermore, one may argue that the low score of the threat is reasonable because the illusory owned body is invisible and the space where the knife cuts is empty. In Experiment 2, however, we did not find any difference between the invisible body and the visible body. The participants perceived the invisible body as being interpolated between gloves and socks, similar to the illusory contour or the amodal completion phenomena^41^. Thus, invisibility cannot account for the low feelings toward the threat. In a further study, as another measure of the startle response, physiological measures^5,29,30^ such as skin conductance response^5^ or event-related cortical potentials^29^ should be employed to clarify these findings.

One may argue that the first-person perspective of the virtual body should be used instead of the third-person perspective (rear view of own body). In experiments for illusory body ownership from the first-person perspective, participants are looking down at their own body and/or looking at a mirror placed in front of them. However, in our preliminary observations, we could feel illusory body ownership to the invisible body from the socks and gloves both from the first-person view and from a viewpoint behind the invisible body. By using the latter, we can measure the proprioceptive self-localization drift. Thus, we adopted the third-person perspective (rear view of own body).

We found that the proprioceptive self-localization drifted to the invisible body area ahead of the participant. This result supports the conclusion that illusory body ownership occurs owing to the invisible body being interpolated between gloves and socks, as do the results of the subjective ratings. Recently, it has been reported that the size perception of external objects is modulated by changing the size of the illusorily owned invisible body^21^. Therefore, we should try to conduct similar experiments on size perception by using our visual-motor active method.

We showed that visual hands and feet are enough to induce illusory body ownership. However, it is unclear whether hands and feet are a minimal or necessary condition for body ownership. This is a limitation of our study and should be investigated in a future study to understand the cognitive mechanism of body ownership.

The illusion of full-body ownership is useful to investigate the idea of minimal phenomenal selfhood for understanding self-consciousness. Virtual-reality techniques enable illusory body ownership to be more flexible. For example, the strength of the body ownership illusion decreases when the virtual body is more transparent, while the pain sensitivity increases as the strength of body ownership in the semi-transparent condition increases^30^. The feeling of ownership of a virtual arm and its vicarious agency were decreased by the visual discontinuity of the arm for both static and dynamic postures^31^. However, we did not find a significant difference between the gloves and socks condition and the whole body condition. These contradictory results may be owing to the difference between a discontinuity or relatively small gap in a body part for body-part ownership and the empty space between hands and feet for full-body ownership, but we need further study in the future.

Relevant to the present study, we may be able to identify the minimal or necessary condition of the Full-Body Illusion or the border between the Full-Body Illusion and the body-part ownership illusion by visual-motor synchronicity. Neural mechanisms of body-part ownership and full-body ownership seem different^1^. We presented only the gloves (hands) and socks (feet) as body parts, but obtained the Full-body Illusion. If we can identify the border between the Full-Body Illusion and the body-part ownership illusion and manipulate it without varying the visual stimuli by using a simple experimental parameter, the experimental paradigm would contribute to clarify the difference in neural mechanisms by combining it with a brain imaging technique in future research.

## Methods

### Experiment 1

#### Participants

Twenty naïve volunteers (all male, mean 21.9 years old ± 0.91 standard deviation (SD)) participated in Experiment 1. They were recruited using posters placed on walls in the Toyohashi University of Technology, and by an announcement made in an undergraduate course lecture ‘Human Information Processing’ of the university irrespective of course credit. All participants were undergraduate or graduate students of Toyohashi University of Technology. All participants for all experiments gave written informed consent, and had healthy vision and were physically healthy. All experiments were approved by the Ethical Committee for Human-Subject Research at Toyohashi University of Technology, and all experiments were performed in accordance with the committee’s guidelines and regulations.

#### Apparatus

Visual stimuli were presented by an HMD (Oculus Rift DK2, 1920 × 1080 pixel, 90 × 110-degree field of view, refresh rate 75 Hz), and appropriately updated with the observer’s head motion. Head-tracking was 6 degrees of freedom. Yaw, roll, and pitch of participants’ heads were sensed by a gyro sensor embedded in the HMD (sampling rate 1 kHz). Positions of the head (x, y, z) were sensed by an optical motion sensor (Microsoft Kinect v2; sampling rate 30 Hz, 512 × 424 pixel resolution). The optical motion sensor also captured the participants’ body movements. A computer (DELL XPS 8700, OS: MS-Windows 8.1, RAM: 16.0 GB, CPU: Intel Core i7-4790 @ 3.60 GHz, GPU: AMD Radeon R9 270) controlled the stimuli and motion sensor.

#### Stimuli and conditions

**Figure 4:**
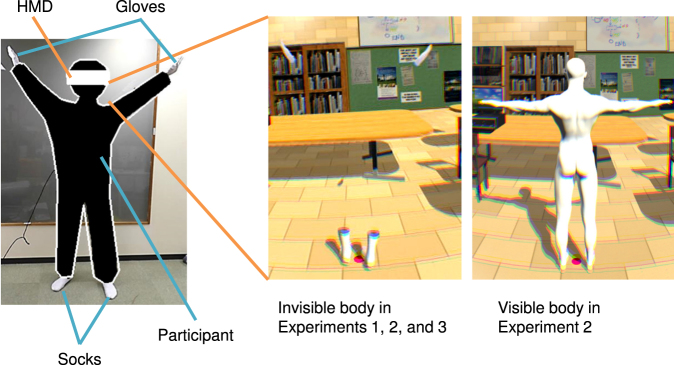
Schematic of the visual-motor synchronous stimuli. (Left) Participants wore white gloves and white socks, and moved freely. (Center) White gloves and white socks were presented as the invisible body stimuli in Experiments 1–3 using an HMD. (Right) A whole body avatar was presented in the visible body condition in Experiment 2.

Participants observed visual motions of white gloves and socks 2 m in front of and facing away from them in a virtual room (Fig. 4). There was no virtual body in their actual body position. They actually put on white gloves and socks during the experiments. The stimuli were presented either synchronously or asynchronously with the observer’s actions in real time. In the synchronous condition, the gloves and socks moved synchronously with participant’s hands and feet motions. However, there was a system delay of approximately 80 ms and the spatial discrepancy (error) was within 10 cm. In the asynchronous condition, the stimuli were replayed from recordings of another person’s actions.

#### Procedures

Participants observed virtual white gloves and socks through the HMD, while they moved their arms and legs freely for 5 min. Then, a knife intended to stimulate the startle response appeared and rotated to cut between the gloves and socks. Participants were asked to answer a questionnaire after each trial to evaluate the illusory body ownership. Each participant performed four trials (2 conditions × 2 repetitions) in either SAAS (S: synchronous condition, A: asynchronous condition) or ASSA order. Thus, the experiment was conducted in within-group design.

In the questionnaire, participants were asked to rate eight items on a seven-level Likert scale ranging from −3 (I did not feel that at all) to 3 (It felt extremely strong) after observing the virtual scene.

1. It felt as if the space between the socks and gloves was my body.
2. It felt as if my body was drifting toward the space between the socks and gloves.
3. It seemed as if I might have more than one body.
4. It felt as if my body became transparent.
5. It seemed as if there is a whole body between the socks and gloves.
6. The movement of the socks and gloves seemed to be my movement.
7. The movement of the socks and gloves seemed to be another person’s movement.
8. It felt as if I was cut by a knife.

### Experiment 2

#### Participants

Twenty naïve volunteers (all male, mean 22.55 years old ± 1.36 SD) participated in Experiment 2. None of them participated in Experiment 1.

#### Stimuli and conditions

Apparatus was identical to Experiment 1. There were two conditions: the invisible body condition and visible body condition (see Fig. 4). The invisible body condition was identical to the synchronous condition in Experiment 1. In the visible body condition, a whole-body avatar was presented and moved synchronously with the participant’s actions, in front of and facing away from the participants similarly to the invisible condition. We chose an adult male model in solid white as the whole-body avatar because the participants were all male adults, and its color was identical to the socks and gloves in the invisible condition.

#### Procedures

The stimulus for the startle response was changed to the colliding motion of a table because it was more natural. After 5 min observation for each trial, a similar questionnaire as in Experiment 1 was used to evaluate the illusory body ownership. Each participant performed four trials (2 conditions × 2 repetitions) in either IVVI (I: invisible condition, V: visible condition) or VIIV order (within-group design).

In the questionnaire, participants were asked to rate eight items on a seven-level Likert scale ranging from −3 (I did not feel that at all) to 3 (It felt extremely strong) after observing the virtual scene. Q3 and Q8 were changed from Experiment 1.

1. It felt as if the space between the socks and gloves was my body.
2. It felt as if my body was drifting toward the space between the socks and gloves.
3. It seemed as if my body became a floor.
4. It felt as if my body became transparent.
5. It seemed as if there is a whole body between the socks and gloves.
6. The movement of the socks and gloves seemed to be my movement.
7. The movement of the socks and gloves seemed to be another person’s movement.
8. It felt as if I collided with the table.

### Experiment 3

#### Participants

Ten volunteers (all male, mean 22.2 years old ± 0.87 SD) who participated in Experiment 1 participated in Experiment 3.

#### Stimuli and conditions

Stimuli and conditions were identical to Experiment 1 except for the control trials. We added two control trials that presented only a virtual room without gloves and socks at the beginning and end of the experiment.

#### Procedures

After observing the stimuli for 5 min, the participants’ proprioceptive self-location was measured. The participants in a black scene of the HMD were moved backward by the experimenter immediately after observing the stimuli in the manner identical to that of Lenggenhager *et al*.^7^. The moving distance was random, between 2.5 m to 3.5 m. The participants were asked to actually walk and return to the original position where they were observing the virtual scene. In the walking return period, the scene in the HMD remained black. Thus, this task was performed without vision. Each participant performed two control trials (the beginning and the final trials), and four experimental trials (2 conditions × 2 repetitions) in ether SAAS (S: synchronous condition, A: asynchronous condition) or ASSA order. Thus, there were in total six trials (within-group design). In the control trials that were conducted before and after the experimental trials, participants performed the self-localization task after observing the identical room without the socks or gloves by moving their body for 5 min. The self-location data measured in the synchronous and asynchronous conditions were subtracted by the self-location data in the control condition (calibration).

### Data Availability

The data are available in supplementary data sheet (S1).

## Electronic supplementary material

Sheet 1
